# A reason why the *ERBB2 *gene is amplified and not mutated in breast cancer

**DOI:** 10.1186/1475-2867-9-5

**Published:** 2009-02-18

**Authors:** Daniel Birnbaum, Fabrice Sircoulomb, Jean Imbert

**Affiliations:** 1 Centre de Recherche en Cancérologie de Marseille, UMR891 Inserm and Institut Paoli-Calmettes, Marseille, F-13009, France; 2 Université de la Méditerranée, Marseille, Cedex 09, F-13288, France; 3 U928 Inserm, TAGC, Marseille-Luminy, France

## Abstract

Alterations of receptor-type tyrosine kinases (RTK) are frequent in human cancers. They can result from translocation, mutation or amplification. The ERBB2 RTK is encoded by a gene that is amplified in about 20% breast cancers. The question is: why is this RTK specifically subjected to this type of alteration? We propose that *ERBB2 *gene amplification is used to overcome repression of its expression by sequence-specific transcription factors.

## Background

Receptor-type tyrosine kinases (RTK) are major regulators of cellular processes. As such they are often mutated in human cancers. Several types of alterations have been characterized. Translocations, amplifications and mutations affect many *RTK *genes in various types of tumors. One of the earliest reports of *RTK *alteration in human cancer was issued more than twenty years ago. It described the amplification of the *ERBB2 *RTK gene in a good proportion of breast cancers [1]. This initial discovery launched the search for other *RTK *alterations in human tumors. This still ongoing search has registered a recent success in neuroblastoma. The *ALK RTK *gene, which is translocated and fused to various partner genes in lymphomas and non-small cell lung cancer [2], has been found amplified and mutated in neuroblastoma [3-6]. While there is no doubt that *RTK *alterations are central to many malignant diseases such as thyroid, lung and breast cancers, a major question remains: what determines the mechanism of alteration (fusion, amplification or mutation) of an *RTK *oncogene?

## Hypothesis

Translocation with fusion may be necessary to both activate the tyrosine kinase and express the oncogenic enzyme in a given tissue or cell. The partner gene will provide the appropriate promoter, dimerization motifs and protein subcellular localization. Mutation is an obvious way of constitutive activation of a kinase. Amplification of the mutated gene, as observed for *ALK *in neuroblastoma, enhances this effect. But why are some *RTK *genes such as *ERBB2 *amplified without mutation or rearrangement? We would like to propose an explanation.

A series of recent works has shed new light on the regulation of *ERBB2 *in mammary epithelial cells. Expression of this gene is apparently tightly controled by a number of transcriptional repressors. FOXP3 represses *ERBB2 *expression, and acts as a tumor suppressor when inactivated [7]. Similarly, PAX2 mediates estrogen receptor (ER) induced repression of *ERBB2 *[8]. We recently found that GATA4 is also a repressor of *ERBB2 *expression [9]. The ETS family member PEA3 can also act as an ERBB2 inhibitor [10]. The Y-box transcription factor CSDA/ZONAB represses *ERBB2 *in a cell-density-dependent manner [11]. Finally, it has been known for long that MYB, which is expressed in ER-positive cells, represses *ERBB2 *[11]. Thus, at least six transcription factors acting as transcriptional repressors of the *ERBB2 *gene have been described so far. It remains to determine whether some, if not all, of these sequence-specific DNA-binding proteins share a common cofactor such as the CTBP corepressor [12], and how many such repressors control the *ERBB2 *promoter.

**Figure 1:**
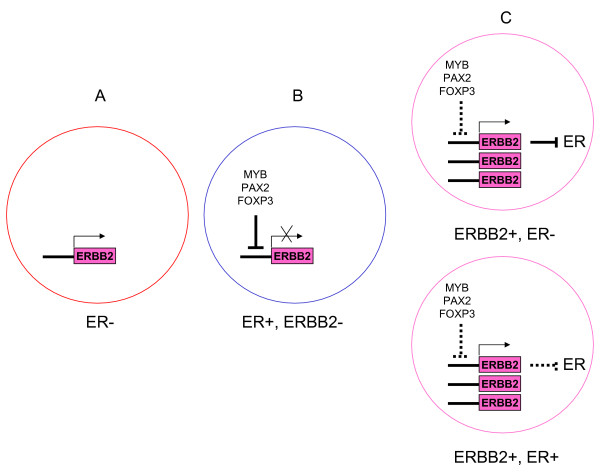
**Schematic representation of the repression/amplification model in mammary epithelial cells**. In these cells *ERBB2 *expression is regulated. A crosstalk between ER and ERBB2 pathways is an important feature of these cells. *ERBB2 *is normally expressed at low level in ER-negative cells (A). In ER-positive cells, *ERBB2 *expression is quenched by ER-induced transcriptional repressors (B). In ERBB2-positive tumors, amplification titrates out the repressors and allows overexpression of *ERBB2*; this may in turn shut down ER expression (C).

The central role of ERBB2 in mammary gland biology makes it a frequent target of mammary oncogenesis. The ERBB2 protein is active in some types of mammary epithelial cells at given periods of development and differentiation but it must be low or absent in ER-positive differentiated cells. The progenitors of these cells should not proliferate outside these regulated periods of mammary gland activity. In these cells *ERBB2 *by-default expression is quenched by strong repressors. We hypothesize that amplification of the *ERBB2 *gene and its cognate non-coding regulatory sequences titrates out these repressors, uncovering a permanent proliferative effect of the ectopically-expressed ERBB2 protein in the progenitors of ER-positive cells. ERBB2 overexpression could in turn shut down more or less tightly ER expression (Figure 1), through the MTA1 repressor [13], the NFKB pathway [14] or other means. When ocurring in ER-negative progenitors, amplification of *ERBB2 *could overcome other repressors that just maintain the necessary low level of expression of *ERBB2 *mRNA, hence increasing the amount of receptor protein up to pathological level.

Other *ERBB2 *repressors remain probably to be discovered, possibly within the list of numerous transcription factors specifically expressed in ER-positive cells [15]. Because of these many and strong repressors, an *ERBB2 *mutated gene would still be repressed and a cell carrying an *ERBB2 *mutation could not be selected. Thus, gene mutation would not be an efficient way to activate ERBB2.

## Testing the hypothesis

Previous works [7] have provided so solid a ground to the hypothesis that it may almost be considered proven. They have shown that overexpression of repressors suppress *ERBB2 *mRNA expression in *ERBB2*-amplified cells. Systematic test of many candidate repressors could be done. There are a number of other relatively easy experiments that would further validate the hypothesis, such as testing if increased expression of *ERBB2 *promoter sequences in mammary epithelial cells, mimicking amplification, would titrate out repressors.

Close examination of the genome of ERBB2 tumors will give us much information. Loss of function of a repressor locus (or several cooperative loci) by deletion or mutation may lead to *de novo ERBB2 *expression and synergize with amplification. FOXP3 mutations have indeed been observed in breast cancer cells [7]. One can also imagine that mutations in the *ERBB2 *promoter region that would remove a repressor binding site may have an effect on *ERBB2 *expression. A good proportion of tumors that overexpress *ERBB2 *do not display *ERBB2 *gene amplification; these tumors could have such loss or mutation. The sequencing of whole tumor genomes will soon tell us if this is the case.

## More general implications

*RTK *genes can display different oncogenic alterations. Titration of sequence-specific repressors or corepressors could be the mechanism at stake in other cases of *RTK *amplification. It could also take place in cases of non-*RTK *gene amplification without mutation. However, in many of these cases (e.g. cyclin D1 or cyclin E in breast cancers), amplification may be the alteration of choice simply because the oncogenic product is the overexpressed normal protein and mutation will not do. Acquisition of a new promoter by translocation and gene fusion would also free an *RTK *oncogene from its natural repressors but has different effects from amplification; it could modify signaling pathways and/or target different cells; in addition constitutive dimerization and activation could bypass other regulatory controls.

In the same line of reasoning, accumulation of gene copies could be a mechanism to escape negative control by microRNAs or any other type of inhibition. For example, amplification might also titrate out methylases to turn on the *ERBB2 *promoter.

The biology of transcriptional repressors will have clinical use. First, knowledge of repressor status may help prognosis assessment and selection of patients for appropriate treatment [8]. Second, transcription could be tightly linked to the development of new therapies. The control of *ERBB2 *gene expression by specific drugs could synergize with anti-receptor or anti-kinase therapy. It should aim at restoring *ERBB2 *repression or inhibiting *ERBB2 *transcription [16,17]. Preclinical trials have used with success expression of PEA3 on mouse xenografts [10]. Chimeric ETS proteins have also shown *ERBB2 *downregulating activity in cell lines [18]. A clinical trial using intratumoral delivery of adenovirus E1A, which can also repress *ERBB2 *expression, has been launched in breast cancer [19]. Members of the FOX family of transcription factors, some of which interact both with *ERBB2 *and ER, are emerging as promising therapeutic targets [20]. Large-scale screens of natural or chemical drugs that modulate *ERBB2 *expression and its interplay with ER could yield interesting molecules [21,22]. A better knowledge of *ERBB2 *promoter and associated transcription factors will probably help find new targets and design new strategies.

The mechanism of oncogenesis involving an RTK may give a clue as to what kind of cell is targeted. A mutated RTK may trigger oncogenic transformation in a cell where it is normally expressed, using the same signaling pathway but in a permanent fashion. An amplified RTK could trigger oncogenesis in a cell (in the case of *ERBB2 *it could be an ER-positive progenitor cell) where it is normally repressed if the reason for the amplification is to remove transcriptional repression, or in a cell where it is normally expressed if the reason is to rise the level of protein made. Amplification of other *RTK *genes such as *EGFR, FGFR1, FGFR2 *and *IGF1R *occurs in various subtypes of breast cancers [23]. It will be interesting to determine whether these genes are also under transcriptional repression and if a general mechanism of oncogenesis associates loss of transcription repressors acting as tumor suppressors and gain of signaling molecules acting as oncogenes.

ERBB2 is associated with stem cell biology in the mammary gland and breast cancer [24]. It would be interesting to determine the level of repressors in breast stem cells and progenitors. Modulation of these repressors may play a role in self-renewal and differentiation.

Finally, the titration of ERBB2 repressors by *ERBB2 *promoter amplification may free other genes from repression by the same transcription factors. *ERBB2 *amplification would thus have consequences outside the activated signaling pathway of the receptor itself. Some of these "liberated" genes might be found upregulated in gene expression analyses of *ERBB2*-amplified tumors [25]. This may explains at least in part why *ERBB2 *amplification is associated with a *bona fide *breast cancer subtype.

## Competing interests

The authors declare that they have no competing interests.

## Authors' contributions

The hypothesis came to view during discussions between the three authors.
